# Introducing a new journal on Parkinson’s disease

**DOI:** 10.1038/npjparkd.2015.6

**Published:** 2015-04-22

**Authors:** David Sulzer, K Ray Chaudhuri, Stanley Fahn

**Affiliations:** 1 Departments of Psychiatry, Neurology and Pharmacology, Columbia University, New York, NY, USA; 2 National Parkinson Foundation International Centre of Excellence, King's College London, London, UK; 3 DeNDroN/Nervous System, London South CRN, King's College Hospital and University Hospital Lewisham, London, UK; 4 Movement Disorders Society Non Motor Study Group, Denmark Hill Campus, London, UK; 5 Department of Neurology, Columbia University, New York, NY, USA; 6 Movement Disorder Division, Neurological Institute, New York, NY, USA

It gives us great pleasure to welcome readers to the first open-access journal from Nature Publishing Group focused on Parkinson’s disease (PD). *npj Parkinson’s Disease* comes online just as we celebrate the publication of the ‘Essay of the Shaking Palsy’ by James Parkinson nearly 200 years ago. Parkinson was an astute clinician who observed the tremor, abnormal gait, slowness and stooped posture of those with the condition that now bears his name. This journal aims to offer its readers other important new information and ideas on this disorder. The journal has been launched in partnership with the Parkinson’s Disease Foundation (PDF), a foremost research- and patient-based organization, and underpins the exciting new concept of journals that are now hosted online and available to any reader, virtually anywhere in the world—for free.

In the past 60 years since the founding of the PDF much has happened in the field of PD: there is effective treatment for many troublesome motor symptoms, with PD being the only neurodegenerative condition with a well-defined neurotransmitter deficiency underpinned by the five decades long success of levodopa therapy. In addition, we have witnessed advances in genetics, molecular biology, stereotactic surgery, and new horizons in therapy, as well as recognition of the key impacts of the non-motor aspects of PD. However, the list of ‘unmet needs’ in PD care and research has also continued to grow. This includes the fact that we do not have adequate animal models that replicate disease progression nor the specific neurons targeted in PD. To date, there are no proven effective treatments for some of the disabling motor and non-motor symptoms of PD, there are no proven neuroprotective treatments to prevent further decline, there are no means to reverse or cure the disease, and we still do not have a clear concept of the pathogenesis of this disorder.

As editors, our not-so-modest goal is to contribute to hastening advances in understanding PD by providing a home where the results of the latest research on the disorder can be shared. We aim to focus on cutting edge research on both care as well as the science of PD, in particular, addressing these unmet needs. We will highlight work from leading workers related to key advances in basic science, genetics, and both motor and non-motor aspects of PD, as well as clinical trials of therapies, interventions, and multidisciplinary care in PD. The open access mechanism of this journal will allow and empower scientists, clinicians, and allied health specialists to provide new information on PD to a global community. A novel feature will be that lay summaries will accompany each article and thus attempt to ‘make simple’ and provide even complex research papers in a ‘user friendly’ format to all readers, particularly patients. The journal will thus serve not only the scientific community but also, equally importantly, people with PD and their care partners—empowering them with access to information that matters and is relevant to the struggle of living with PD.

